# Soybean-derived Bowman-Birk Inhibitor (BBI) Inhibits HIV Replication in Macrophages

**DOI:** 10.1038/srep34752

**Published:** 2016-10-13

**Authors:** Tong-Cui Ma, Run-Hong Zhou, Xu Wang, Jie-Liang Li, Ming Sang, Li Zhou, Ke Zhuang, Wei Hou, De-Yin Guo, Wen-Zhe Ho

**Affiliations:** 1 Wuhan University School of Basic Medical Sciences, Wuhan, Hubei, 430071, P.R. China; 3 Department of Pathology and Laboratory Medicine, Temple University Lewis Katz School of Medicine, Philadelphia, PA 19140, USA; 2 State Key Laboratory of Virology, Wuhan University, Wuhan, Hubei, 430071, P.R. China

## Abstract

The Bowman-Birk inhibitor (BBI), a soybean-derived protease inhibitor, is known to have anti-inflammatory effect in both *in vitro* and *in vivo* systems. Macrophages play a key role in inflammation and immune activation, which is implicated in HIV disease progression. Here, we investigated the effect of BBI on HIV infection of peripheral blood monocyte-derived macrophages. We demonstrated that BBI could potently inhibit HIV replication in macrophages without cytotoxicity. Investigation of the mechanism(s) of BBI action on HIV showed that BBI induced the expression of IFN-β and multiple IFN stimulated genes (ISGs), including Myxovirus resistance protein 2 (Mx2), 2′,5′-oligoadenylate synthetase (OAS-1), Virus inhibitory protein (viperin), ISG15 and ISG56. BBI treatment of macrophages also increased the expression of several known HIV restriction factors, including APOBEC3F, APOBEC3G and tetherin. Furthermore, BBI enhanced the phosphorylation of IRF3, a key regulator of IFN-β. The inhibition of IFN-β pathway by the neutralization antibody to type I IFN receptor (Anti-IFNAR) abolished BBI-mediated induction of the anti-HIV factors and inhibition of HIV in macrophages. These findings that BBI could activate IFN-β-mediated signaling pathway, initialize the intracellular innate immunity in macrophages and potently inhibit HIV at multiple steps of viral replication cycle indicate the necessity to further investigate BBI as an alternative and cost-effective anti-HIV natural product.

As one of the primary targets for HIV infection and persistence, macrophages have been indicated as an important HIV reservoir for viral latency. In addition, macrophages activation contributes to HIV-mediated inflammation, as they can release inflammatory cytokines that induce systemic immune activation. Studies have clearly shown that chronic immune activation and inflammation are associated with CD4^+^ T cell depletion and HIV disease progression^1^,^2^,^3^,^4^,^5^,^6^,^7^. Conversely, macrophages play an important role in the host defense against HIV infection. Macrophages produce the multiple intracellular HIV restriction factors^8^,^9^. HIV-infected macrophages produce viperin which suppresses viral replication through the internal S-adenosyl methionine domains of viperin^9^. Macrophages also express tetherin (BST-2/CD317/HM1.24) that has the ability to block HIV release from infected cells^8^. Our early study showed that TLR3 activation of macrophages potently suppresses HIV infection and replication through multiple antiviral mechanisms at both cellular and molecular levels^10^. As HIV latency is the major obstacle in preventing the eradication of the viruses, it is crucial to identify agents that can activate intracellular innate immunity against HIV in the target cells, such as macrophages.

Serine proteases are known to be actively involved in pro-inflammatory actions^11^, including the production of inflammatory cytokines, including TNF-α, IL-1β, IL-6, which enhance HIV infection^12^,^13^,^14^,^15^,^16^. Bowman-Birk inhibitor (BBI) is a serine proteases inhibitor^11^. BBI is present in many commercial soy foods, such as soymilk, soy-based infant formula, and bean curd. BBI has been shown to have anti-inflammatory effect in both *in vitro* and *in vivo* systems^11^,^17^,^18^,^19^,^20^. BBI exerts its immunoregulation function through inhibition of proteases released from inflammation-mediating cells^21^. BBI reduces autoimmune inflammation and attenuates neuronal injury^22^. Safavi *et al*. demonstrated the immunoregulatory and anti-inflammatory effects of BBI in the experimental autoimmune encephalomyelitis (EAE) model^23^. BBI administration could delay the onset of EAE and reduced its severity in an IL-10-dependent manner^24^. The importance of BBI in the regulation of inflammation is highlighted by its ability to decrease LPS-induced inflammatory cytokines (TNF-α, IL-1β, IL-6) and increase anti-inflammatory cytokine (IL-10) in macrophages^19^. In addition, BBI treatment could increase proportion of CD4^+^CD25^+^Foxp3^+^ Tregs^24^ that have potent immune-suppressive activities^25^. Because macrophage-mediated inflammation and/or immune activation have the contributing roles in the immunopathogenesis and progression of HIV disease, we sought to determine the effect of BBI on HIV infection of macrophages. We also explored the mechanisms involved in the BBI action on HIV.

## Results

### BBI inhibits HIV infection of macrophages

**Figure 1:**
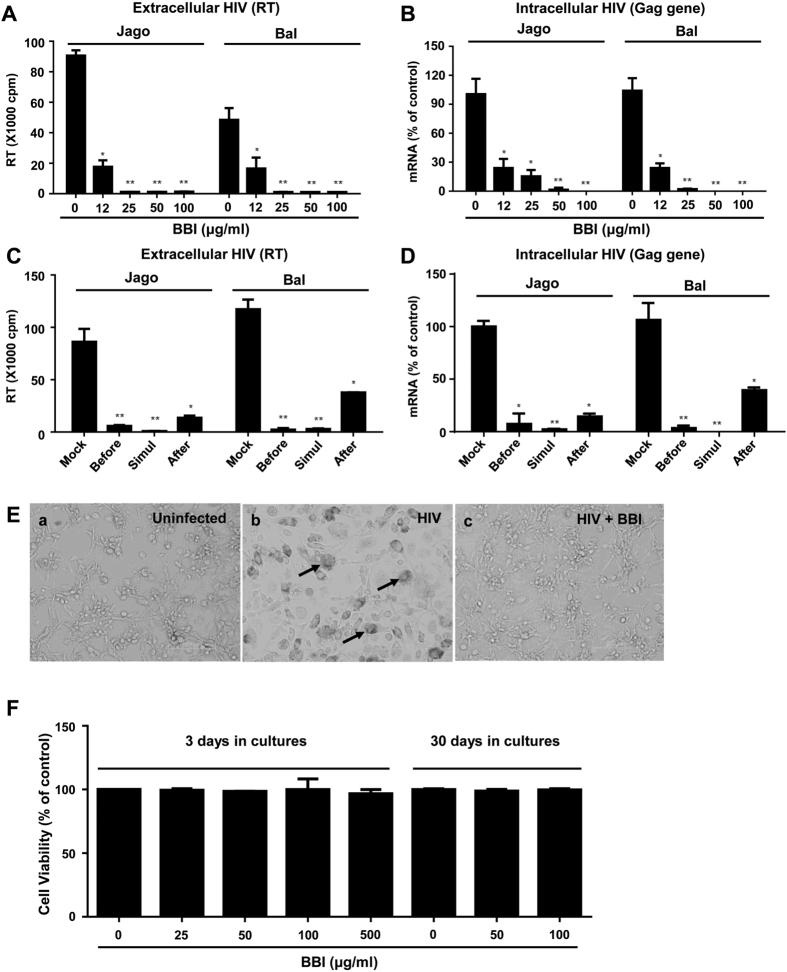
BBI inhibits HIV infection of macrophages. Peripheral blood monocyte-derived macrophages were treated with/without indicated concentrations of BBI for 24 h prior to HIV Jago or Bal infection. After washing away unbound virus, fresh medium containing corresponding doses of BBI was added to the cultures. **(A)** Cell-free supernatant was collected on day 12 post-infection for HIV Reverse transcriptase (RT) assay. **(B)** Cellular RNA was subjected to real time RT PCR for HIV Gag and GAPDH RNA. Macrophages were treated with BBI (100 μg/ml) either 24 h prior to HIV (Bal strain) infection (Before), simultaneously with infection (Simultaneously), or 48 h post-infection (After). Macrophages cultured in the presence of BBI for 12 days. **(C)** Cell-free supernatant was collected on day 12 post-infection for HIV Reverse transcriptase (RT) assay. **(D)** Cells were harvested on day 12 and cellular RNA was subjected to real time RT PCR for HIV Gag and GAPDH RNA. Data are expressed as HIV RNA levels percentage (%) to untreated control, which is defined as 100% (Simul: Simultaneously). **(E)** The effect of BBI pretreatment of macrophages on HIV-induced syncytium formation. The morphology of untreated and uninfected, untreated and HIV-infected (Bal strain), BBI-pretreated (100 μg/ml) and HIV-infected macrophages was observed, the images were obtained under a light microscope (magnification, ×200) on day 12 post-infection. The arrows indicated HIV-induced giant syncytia. **(F)** The cytotoxicity effect of BBI on macrophages. Seven-day-cultured macrophages were treated with/without BBI at indicated concentrations for 3 days or 30 days. The cell viability was assessed by MTS assay. Data are showed as the absorbance (490 nm) relative to untreated control, which is defined as 1.0. The results shown in (**A–F**) were obtained as mean ± SD from three independent experiments with triplicate wells (*P < 0.05, **P < 0.01).

To determine anti-HIV effect of BBI, seven-day-cultured macrophages were pretreated with different concentrations of BBI (12, 25, 50, and 100 μg/ml) and followed by HIV (Jago and Bal) infection. As shown in Fig. 1, both extracellular HIV RT activity (cell-free supernatant) (Fig. 1A) and intracellular HIV Gag gene expression (Fig. 1B) were inhibited by BBI. This inhibitory effect of BBI (the dose of 25 μg/ml or higher) on HIV strains Jago and Bal was highly significant with IC50 of 6.57 μg/ml and 10.18 μg/ml, respectively. To further determine the anti-HIV ability of BBI, macrophages were treated with BBI under different treatment conditions (before, simultaneously with, or after HIV Bal infection). BBI treatment of macrophages during HIV Bal infection (simultaneously) was the most effective in viral inhibition at both extracellular (Fig. 1C) and intracellular (Fig. 1D) levels. Macrophages treated with BBI before or after HIV Bal infection also significantly suppressed viral expression (Fig. 1C,D). Morphologically, HIV Bal-infected macrophage cultures without BBI treatment demonstrated characteristic giant syncytium formation (Fig. 1E, panel b), whereas BBI-treated macrophages failed to develop HIV-induced giant syncytia (Fig. 1E, panel c). To determine whether the anti-HIV effect of BBI is not associated with cytotoxicity, we examined whether BBI is toxic to macrophages. As shown in Fig. 1F, there was no cytotoxicity of BBI on macrophages.

### BBI induces the phosphorylation of IRF3 and up-regulates IFN-β

**Figure 2:**
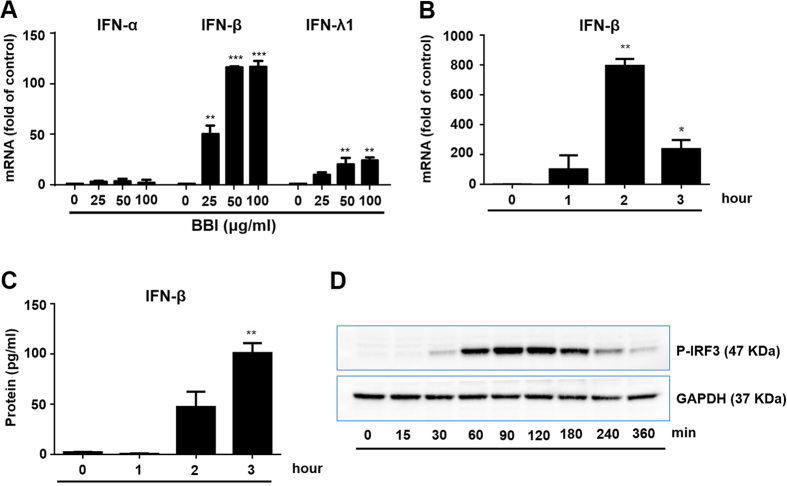
BBI induces the phosphorylation of IRF3 and increases the expression of IFN-β. **(A)** Peripheral blood monocyte-derived macrophages were treated with/without BBI at indicated concentrations for 2 h. Cellular RNA was collected and subjected to real time RT PCR for IFN-α, IFN-β, IFN-λ1 and GAPDH mRNA. Data are expressed as genes levels relative (fold) to untreated control, which is defined as 1.0. **(B)** Macrophages were treated with/without 50 μg/ml BBI for indicated times. Cellular RNA was collected and subjected to real time RT PCR for IFN-β and GAPDH mRNA. Data are expressed as genes levels relative (fold) to untreated control, which is defined as 1.0. **(C)** Macrophages were treated with/without BBI at 50 μg/ml for indicated times. Cell-free supernatant was collected and subjected to ELISA. **(D)** Macrophages were treated with/without BBI at 50 μg/ml for indicated times. Cellular protein was collected and subjected to Western blot. Representative data from three independent experiments are shown. The results shown in (**A–C**) were obtained as mean ± SD from three independent experiments with triplicate wells (*P < 0.05, **P < 0.01, ***P < 0.001).

To understand the mechanism(s) of BBI-mediated HIV inhibition, we examined the effect of BBI on IFN expression in macrophages. As shown in Fig. 2A–C, BBI selectively upregulated the expression of IFN-β at both mRNA and protein levels. Furthermore, BBI treatment of macrophages enhanced the phosphorylation of IRF3 (Fig. 2D), a key regulator of IFN-β^26^,^27^.

### BBI enhances the phosphorylation of STAT1 and STAT3

**Figure 3:**
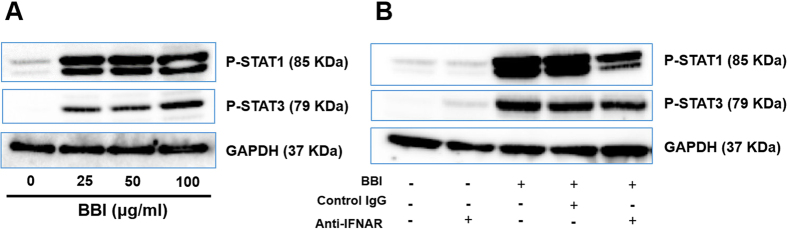
BBI induces the phosphorylation of STAT1 and STAT3. **(A)** Peripheral blood monocyte-derived macrophages were treated with/without BBI at indicated concentrations for 3 h. Cellular protein was collected and subjected to Western blot. **(B)** Macrophages were treated with 10 μg/ml anti-IFNAR or control IgG for 1 h and incubated with 50 μg/ml BBI for additional 3 h. Cellular protein was collected and subjected to Western blot. Representative data from three independent experiments are shown.

Because IFN-β is a key activator of the JAK/STAT pathway^28^,^29^,^30^, we next investigated the effect of BBI on the phosphorylation of STAT1 and STAT3. As shown in Fig. 3A, BBI was able to enhance the phosphorylation of STAT1 and STAT3 in macrophages. To study the role of IFN-β in the BBI actions on the JAK/STAT pathway, we performed the neutralization assays with specific antibodies to IFN-alpha/beta receptor. As shown in Fig. 3B, the neutralization antibody to IFN-alpha/beta receptor (anti-IFNAR) could counteract the BBI-induced phosphorylation of STAT1 and STAT3.

### BBI induces ISGs and HIV restriction factors

**Figure 4:**
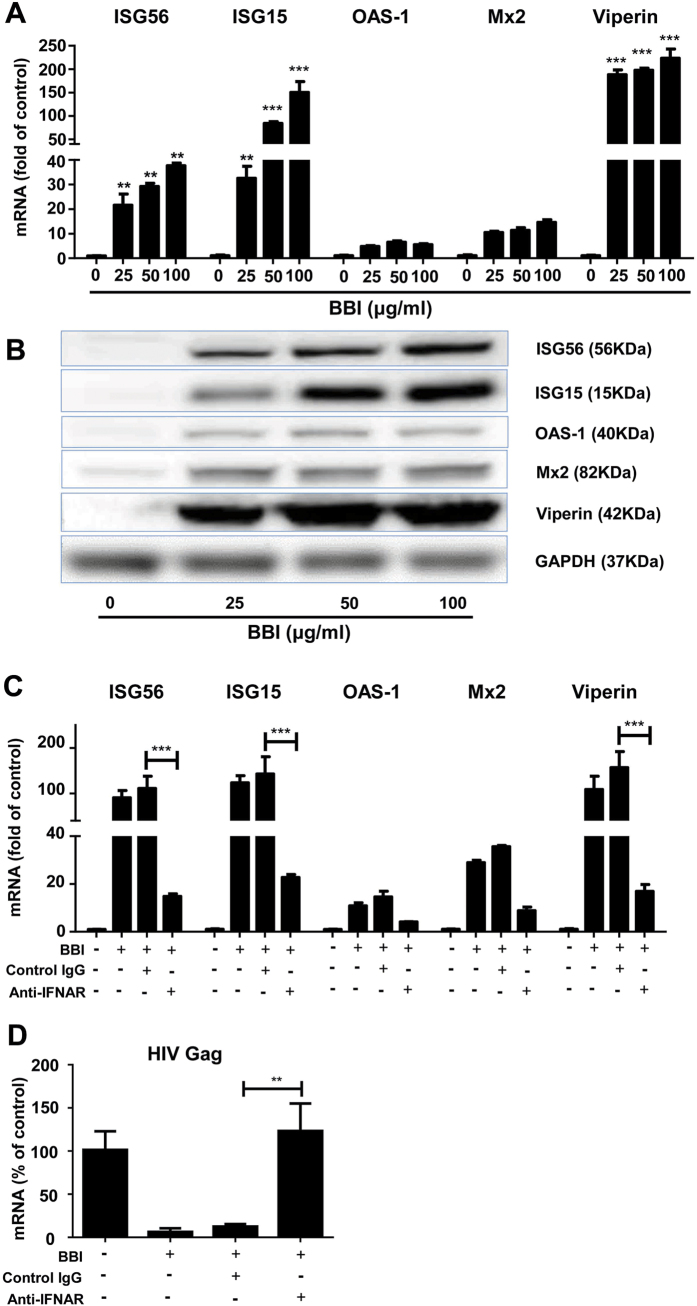
BBI induces ISGs. Peripheral blood monocyte-derived macrophages were treated with/without BBI at indicated concentrations for 6 h or 24 h. **(A)** For mRNA quantification, cellular RNA was collected at 6 h post-treatment and subjected to real time RT PCR for genes indicated and GAPDH RNA. Data are expressed as the levels of tested genes relative (fold) to untreated control, which is defined as 1.0. **(B)** For protein quantification, macrophages were treated with/without BBI at indicated concentrations for 24 h, cellular proteins were collected and subjected to Western blot. Representative data from three independent experiments are shown. **(C)** Macrophages were treated with 10 μg/ml neutralization antibody to IFN- alpha/beta receptor (anti-IFNAR) or control IgG for 1 h and incubated with 50 μg/ml BBI for additional 6 h. Cellular RNA was collected and subjected to real time RT PCR for genes indicated and GAPDH RNA. Data are expressed as genes levels relative (fold) to untreated control, which is defined as 1.0. **(D)** Macrophages were treated with/without 10 μg/ml anti-IFNAR or control IgG for 1 h and incubated with 50 μg/ml BBI for additional 24 h prior to HIV (Bal strain) infection. After washing away unbound virus, fresh medium containing BBI, anti-IFNAR or control IgG was added to the cultures. Cells was collected on day 7 for HIV Gag gene and GAPDH expression. Data are expressed as genes levels relative (%) to untreated control, which is defined as 100%. The results shown in (**A–D**) were obtained as mean ± SD from three independent experiments with triplicate wells (**P < 0.01, ***P < 0.001).

**Figure 5:**
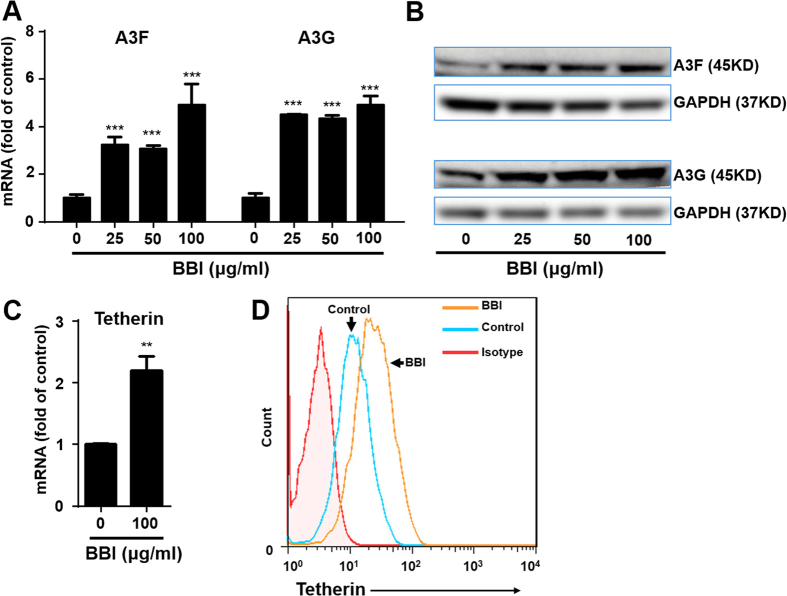
BBI induces APOBEC3F/G and tetherin. Peripheral blood monocyte-derived macrophages were treated with/without BBI at indicated concentrations for 6 h or 24 h. **(A)** For mRNA quantification, cellular RNA was collected at 6 h post-treatment and subjected to real time RT PCR for genes indicated and GAPDH RNA. Data are expressed as genes levels relative (fold) to untreated control, which is defined as 1.0. **(B)** For A3F/A3G expression at protein level, macrophages were treated with/without BBI at indicated concentrations for 24 h. Cellular protein was collected and subjected to western blot. **(C)** Macrophages were treated with/without 100 μg/ml BBI for indicated times. Cellular RNA was collected and subjected to real time RT PCR for tetherin and GAPDH mRNA. Data are expressed as genes levels relative (fold) to untreated control, which is defined as 1.0. **(D)** For tetherin expression at protein level, macrophages were treated with/without BBI (100 μg/ml) for 24 h. Cells were harvested and incubated with PE-tetherin (BST2, CD317) antibody. Unstained or isotype-matched mouse immunoglobulin G-stained cells were included as a negative control. Stained cells were acquired by fluorescence activated cell sorting and analyzed using FlowJo software. Representative data from three independent experiments are shown in (**B,D**). The results shown in (**A,C**) were obtained as mean ± SD from three independent experiments with triplicate wells (**P < 0.01, ***P < 0.001).

IFN stimulated genes (ISGs) and cellular antiviral factors are the key elements in host cell innate immunity against viral infections. We showed that BBI treatment of macrophages significantly induced several antiviral ISGs (ISG15, ISG56, OAS-1, Mx2, viperin) at both mRNA (Fig. 4A) and protein (Fig. 4B) levels. To investigate the role of IFN-β in the BBI actions on the anti-HIV factors and inhibition of HIV, we performed the neutralization assays with specific antibody to IFN-alpha/beta receptor. As shown in Fig. 4C,D, the neutralization antibody to IFN-alpha/beta receptor (Anti-IFNAR) could compromise the BBI-mediated production of anti-viral factors and inhibition of HIV. In addition, BBI also increased the expression of several APOBEC3 family members, particularly APOBEC3G (A3G) and APOBEC3F (A3F). As shown in Fig. 5A,B, BBI induced A3F/A3G at both mRNA and protein levels. Furthermore, BBI treatment of macrophages increased the expression of tetherin at both mRNA (Fig. 5C) and protein (Fig. 5D) levels.

### BBI up-regulates RIG-I and MDA-5

**Figure 6:**
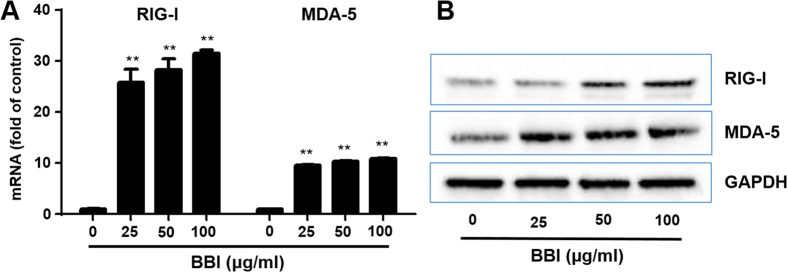
BBI up-regulates RIG-I/MDA-5. **(A)** Peripheral blood monocyte-derived macrophages were treated with/without BBI at indicated concentrations for 6 h. Cellular RNA was collected and subjected to real time RT PCR for the genes of RIG-I, MDA-5 and GAPDH. Data are expressed as genes levels relative (fold) to untreated control, which is defined as 1.0. The results were obtained as mean ± SD from three independent experiments with triplicate wells (**P < 0.01). (**B**) For RIG-I and MDA-5 expression at protein level, macrophages were treated with/without BBI at indicated concentrations for 24 h. Cellular protein was collected and subjected to Western blot. Representative data from three independent experiments are shown.

To further investigate the intracellular mechanism of BBI-mediated induction of anti-HIV innate immunity in macrophages, we examined the effect of BBI on the expression of RIG-I/MDA-5, the key intracellular sensors of viral RNAs that can trigger IFN-mediated immune response to viral replication. BBI could significantly increase the expression of both RIG-I and MDA-5 at the levels as high as 30 and 15 folds, respectively (Fig. 6A). In addition, BBI also induced the expression of both RIG-I and MDA-5 at protein levels (Fig. 6B).

### BBI increases the expression of antiviral factors in HIV-infected macrophages

**Figure 7:**
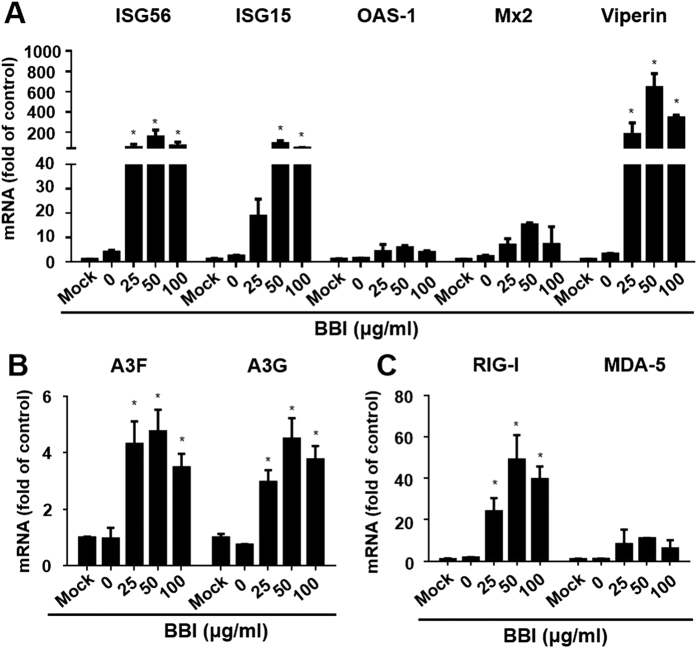
BBI upregulates antiviral factors in HIV-infected macrophages . **(A–C)** Peripheral blood monocyte-derived macrophages were infected with HIV (Bal strain) for 48 h and then incubated with/without BBI at indicated concentrations for 6 h. Cells were collected for the indicated genes and GAPDH expression. Data are expressed as genes levels relative (fold) to untreated control, which is defined as 1.0. The results were obtained as mean ± SD from three independent experiments with triplicate wells (*P < 0.05).

We also examined whether BBI could induce the expression of anti-viral ISGs and HIV restriction factors in HIV-infected macrophages. As shown in Fig. 7A, BBI still up-regulated the expression of anti-viral ISGs (ISG15, ISG56, OAS-1, Mx2, viperin) in HIV Bal-infected macrophages. In addition, BBI treatment of macrophages up-regulated the expression of cellular HIV restriction factors, A3F/A3G, (Fig. 7B) as well as the intracellular viral RNA sensors, RIG-I/MDA-5 (Fig. 7C).

## Discussion

In this study, we demonstrated for the first time that BBI, a natural product from soy foods, could potently inhibit HIV infection of macrophages without cytotoxicity. The inhibitory effect of BBI on HIV was highly effective (>90% inhibition) at dose of 25 μg/ml or higher (Fig. 1A,B). When macrophages were treated with BBI and infected with HIV simultaneously, a nearly complete inhibition of HIV infection of macrophages was observed. This significant inhibitory effect of BBI was also observed before or after HIV infection of macrophages (Fig. 1C,D). To understand the mechanisms by which BBI inhibits HIV, we examined the impact of BBI on IFNs and IFN-induced antiviral factors in macrophages. We found that BBI treatment of macrophages could selectively induce the expression of IFN-β (Fig. 2A) and multiple ISGs (ISG56, ISG15, OAS-1, Mx2 and viperin) (Fig. 4A,B) that have ability against a number of viruses, including HIV. For example, ISG15 has a crucial role in the IFN-mediated inhibition of late stages of HIV assembly and release;^31^ Mx2 is a newly identified IFN-induced inhibitor of HIV infection^32^,^33^,^34^. Mx2 inhibits HIV infection by inhibiting capsid-dependent nuclear import of subviral complexes^33^. In addition, BBI could upregulate the expression of ISGs in HIV-infected macrophages (Fig. 7A). In addition to the ISGs, we found that BBI also induced the expression of several key HIV restriction factors, including APOBEC3F (A3F), APOBEC3G (A3G) (Fig. 5A,B) and tetherin (Fig. 5C,D). A3G/A3F are the single-stranded DNA deaminases that inhibit HIV replication through deaminating cytidine to uracil on the minus strand of the HIV proviral DNA;^35^ Tetherin is a transmembrane protein that specifically inhibits HIV release from infected cells^36^.

One of the important observations of this study is that BBI selectively upregulated IFN-β (Fig. 2A,B). The role of IFN-β in the BBI-mediated induction of the ISGs and the HIV restriction factors is confirmed, as the neutralization antibody to IFN-alpha/beta receptor could abolish the BBI actions (STATs, ISGs, and HIV; Figs 3B and 4C,D). It is well known that type I IFNs control viral infection/replication principally through the upregulation of antiviral ISGs. We demonstrated that BBI is able to induce anti-HIV ISGs, such as ISG15 and Mx2 (Fig. 4A,B) as well as the HIV restriction factors (3G/F and tetherin) (Fig. 5A,B). These factors can inhibit HIV at different steps of the viral replication. However, the role of IFNs and ISGs in HIV pathogenesis and treatment remain to be determined. It was reported that there were deleterious effects of IFN on HIV infection^37^. The investigators showed that the chronically HIV-infected subjects had the hyperactivation of effector cells and subsequent production of IFN/ISGs, which could exert suppressive and cytotoxic effects on T cells^4^. Therefore, further studies on the interplays between HIV and IFN signaling pathway are necessary to guide future intervention and therapeutic strategies.

Because the activation of JAK-STAT signaling pathway is crucial in type I IFN-mediated induction of antiviral factors, we thus investigated the role of BBI in the activation of the JAK/STAT pathway. We found that BBI treatment of macrophages enhanced the phosphorylation of both STAT1 and STAT3 (Fig. 3A). To further understand intracellular immunity involved in the BBI action on HIV inhibition, we examined the impact of BBI on RIG-I and MDA-5. As an intracellular sensor of viral replication, RIG-I plays an important role in host cell innate immunity against viral infections. RIG-I recognizes viral RNA and activates the type I IFN-dependent antiviral innate-immune responses, which is involved in the JAK/STAT signaling pathway^38^. In addition to RIG-I, MDA-5 is another cytoplasmic sensor of intracellular viral RNAs^39^,^40^. We found that BBI significantly induced the expression of both RIG-I and MDA-5 (Fig. 6A,B). We also examined the impact of BBI on the TLRs, as they are crucial in triggering the innate immune responses of host cells to pathogens. It is known that TLR3, 7, 8, 9^41^ recognize and respond to viral infections, inducing IFNs and ISGs. However, we found that BBI had little effect on the expression of these TLRs (data not shown). Although BBI has been extensively used in both *in vitro* and *in vivo* studies, the precise mechanism(s) of BBI entry into cells remain to be determined. Several papers^42^,^43^ reported the possible receptors for BBI entry into cells. However, due to the lack of commercial antibody to BBI receptor, we were unable to determine whether the BBI actions on HIV and the host cell immunity were the receptor-mediated. Because macrophages have the function of phagocytosis, it is possible that BBI may enter macrophages by phagocytosis. Nevertheless, future studies with the specific antibody to BBI or BBI receptor are necessary in order to determine the entry mechanism(s) of BBI in macrophages and other cell systems.

Taken together, we have provided the compelling evidence that BBI potently inhibits HIV infection of macrophages. Given that macrophages are an important cellular reservoir for HIV infection/persistence, to control and eradicate HIV in macrophages is clinically significant. Although the precise cellular and molecular mechanisms by which BBI inhibits HIV replication remain to be determined, the induction of IFN-β, several antiviral ISGs and HIV restriction factors in macrophages should account for much of BBI-mediated anti-HIV activity. These anti-HIV activities of BBI are clinically important and significant, as it is unlikely for HIV to develop resistance to BBI. Given the fact that there is limited access to conventional anti-HIV drugs in developing countries and emergence of resistant mutants of HIV, BBI and related natural products may provide an excellent source for developing novel and cost-effective anti-HIV drugs. Therefore, there is a necessity of future *in vivo* studies for the development of BBI-based supplementary therapy for people infected with HIV, particularly those in resource poor settings.

## Materials and Methods

### Reagents and antibodies

Bowman-Birk inhibitor (BBI) was purchased from Sigma-Aldrich (St. Louis, MO, USA). The product is isolated from Glycine max (soybean) and purified from crude trypsin inhibitor (Sigma Cat #T9128). It consists of 90% protein as assayed by Biuret, with the remainder a phosphate buffer salt. The stock solution was prepared in sterile culture grade water at 1 mg/ml. Rabbit antibodies against ISGs (ISG56, ISG15, OAS-1, viperin), RIG-I/MDA-5, p-IRF3 and p-STAT1/3 were purchased from Cell Signaling Technology (Danvers, MA). Rabbit antibodies against Mx2 was purchased from Novus Biologicals (Littleton, CO). Anti-human interferon alpha/beta receptor chain 2 (anti-IFNAR), clone MMHAR-2 (MAb) (Cat #:21385-1), was purchased from PBL Assay Science (Piscataway, NJ).

### MTS

The impact of BBI treatment on the viability of monocyte-derived macrophages was analyzed by 3-(4, 5-dimethylthiazol-2-yl)-5-(3-carboxymethoxyphenyl)-2-(4-sulfophenyl) -2H-tetrazolium, inner salt (MTS) assay. Macrophages cultured in a 96-well plate were treated with different concentrations of BBI (25, 50, 100, 500 μg/ml) for 72 h or BBI (50, 100 μg/ml) for 30 days. For MTS assay, 20 μl of CellTiter 96^**®**^ AQ_ueous_ One Solution Reagent containing MTS and phenazine ethosulfate was added to each well of the 96-well plate. Absorbance at 490 nm was measured at 4 h after addition of the reagent.

### Macrophages and HIV strains

Purified human peripheral blood monocytes were purchased from Human Immunology Core at the University of Pennsylvania (Philadelphia, PA, USA). The Core has the Institutional Review Board approval for blood collection from healthy donors. Freshly isolated monocytes were cultured in the 48-well plate (2.5 × 10^5^ cells/well) in DMEM containing 10% FBS. Macrophages refer to 7-day-cultured monocytes. The HIV R5 strains (Bal and Jago) were obtained from the AIDS Research and Reference Program of the National Institutes of Health (Bethesda, MD).

### BBI treatment and HIV infection

Macrophages were treated with different concentrations (25, 50, and 100 μg/ml) of BBI either before (24 h), simultaneously or after (48 h) HIV (Jago, Bal) infection. After washing away unattached virus, infected-macrophages were cultured in medium containing corresponding doses of BBI. The productive HIV infection was determined on day 12 post-infection. In the experiments to study the role of IFN-β in the BBI action against HIV, the anti-IFNAR antibody was added to cell cultures 1 h prior to BBI treatment.

### Reverse transcription and quantitative real time PCR

Total RNA from macrophages or cell-free supernatant was extracted using Tri-Reagent (Molecular Research Center, Cincinnati, OH). Reverse transcription was performed using the random primer, dNTP, AMV transcriptase and RNase inhibitor (Promega Co., Madison, WI, USA) according to the manufacturer’s instruction. Quantitative Real-time PCR (qRT-PCR) was performed with Brilliant SYBR Green Master Mix (Bio-Rad Laboratories, Hercules, CA, USA). The primers used for the qRT-PCR amplifications are synthesized by Integrated DNA Technologies (Coralville, IA, USA) and sequences will be available upon request. All values for RNA quantitative from macrophages were calculated using the delta delta Ct method^44^ and expressed as the changes relative to the expression of glyceraldehyde 3-phosphate dehydrogenase (GAPDH) mRNA.

### Reverse transcriptase (RT) assay

HIV RT activity was determined based on the technique of Willey *et al*.^45^ with modifications^46^,^47^. In brief, 10 μl of culture supernatant was added to 50 μl cocktail containing poly A, oligo-dT and (^32^P) dTTP and incubated overnight at 37 °C. Then, the cocktail (30 μl) was spotted onto pre-marked DE81 paper, dried for 15 min, and washed 4 times with 2x saline-sodium citrate buffer and once with 95% ethanol. The filter paper was then air-dried, cut into small pieces and put into bottles which contain 1 ml scintillation liquid. Radioactivity was counted in a liquid scintillation counter.

### ELISA

Cell-free supernatant from macrophage cultures treated with/without different concentrations of BBI (25, 50, 100 μg/ml) for 24 h was collected for the analysis of protein level of IFN-β with ELISA kit from PBL Assay Science (Piscataway, NJ). ELISA was performed according to the manufacturer’s instructions.

### Western blot

With the antibodies available, the expression of the ISGs (ISG15/56, OAS-1, Mx2, viperin), HIV restriction factors (A3F/A3G), RIG-I/MDA-5, phosphorylated-IRF3, and phosphorylated-STATs (p-STAT1 and p-STAT3) were evaluated by Western blot. Following incubation with monoclonal antibodies to the ISGs, RIG-I/MDA-5, p-IRF3 and p-STAT1/3 and extensive washing in PBS containing 0.05% Tween-20, the membranes were incubated with horseradish peroxidase-conjugated IgG (Pierce, Chester, UK) for 1 h at room temperature. The membranes were further washed in PBS. The immunoblots were visualized by enhanced chemiluminescence detection (ECL, Amersham, Bucks, UK).

### Flow cytometric analysis

The expression of tetherin was examined by flow cytometer. Macrophages were detached with versene buffer (8 g NaCl, 0.2 g KCl, 1.15 g Na_2_HPO_4_, 0.2 g EDTA, 0.1 g Phenol Red in 1L). After washing with Phosphate-buffered saline containing 1% fetal bovine serum, macrophages were incubated with PE-tetherin (BST2, CD317) antibody (Biolegend, San Diego, CA) at room temperature for 30 min. Unstained or isotype-matched mouse immunoglobulin G-stained cells were included as a negative control. Stained cells were acquired by fluorescence activated cell sorting (FACSCantoII; BD Bioscience, San Jose, CA) and analyzed using Flow-Jo software (Tree Star Inc, Ashland, OR).

### Statistical analysis

Where appropriate, data were expressed as mean ± standard deviation (SD) of triplicate cultures. Statistical significance was assessed by Student’s t test or Two-way ANOVA. Statistical analyses were performed with Graph pad prism Statistical Software. Statistical significance was defined as P < 0.05.

## Additional Information

**How to cite this article**: Ma, T.-C. *et al*. Soybean-derived Bowman-Birk Inhibitor (BBI) Inhibits HIV Replication in Macrophages. *Sci. Rep.* **6**, 34752; doi: 10.1038/srep34752 (2016).
