# Corrigendum: Soybean-derived Bowman-Birk Inhibitor (BBI) Inhibits HIV Replication in Macrophages

**DOI:** 10.1038/srep37383

**Published:** 2016-12-23

**Authors:** Tong-Cui Ma, Run-Hong Zhou, Xu Wang, Jie-Liang Li, Ming Sang, Li Zhou, Ke Zhuang, Wei Hou, De-Yin Guo, Wen-Zhe Ho

Scientific Reports 6: Article number: 3475210.1038/srep34752; published online: 10 13 2016; updated: 12 23 2016

In the PDF version of this Article, affiliations 2 and 3 are reversed; the HTML version of the Article was correct at the time of publication.

